# Supplementary material for: A Simultaneous Extraction/Derivatization Strategy for Quantitation of Vitamin D in Dried Blood Spots Using LC–MS/MS: Application to Biomarker Study in Subjects Tested for SARS-CoV-2
Source: Int J Mol Sci. 2023 Mar 13;24(6):5489. doi: 10.3390/ijms24065489 (PMC10054002; doi:10.3390/ijms24065489)
Supplement: Supplementary file 1 [file ijms-24-05489-s001.zip › ijms-2210678-supplementary.docx]

**Supplementary Files**

**A simultaneous extraction/derivatization strategy for quantitation of Vitamin D in Dried blood spots using LC-MS/MS: Application to biomarker study in Subjects Tested for SARS-CoV-2**

Yashpal S. Chhonker^1, #^, Nusrat Ahmed^1, #^, Christine M. Johnston^2^, Ruanne Barnabas^3,4^, Daryl J. Murry^1, 5, ∆^

^1^Clinical Pharmacology Laboratory, Department of Pharmacy Practice and Science, University of Nebraska Medical Center, Omaha, NE 68198, United States

^2^Department of Medicine, University of Washington, Seattle, WA

^3^Division of Infectious Diseases, Massachusetts General Hospital, Boston, MA

^4^Harvard Medical School, Boston, MA

^5^Fred and Pamela Buffett Cancer Center, University of Nebraska Medical Center, Omaha, NE

68198, United States.

^#^ Authors contributed equally to this manuscript

^∆^ Corresponding author:

Daryl J. Murry, Pharm. D. (E-mail: [dj.murry@unmc.edu](mailto:dj.murry@unmc.edu))

Clinical Pharmacology Laboratory

Department of Pharmacy Practice and Science, College of Pharmacy

University of Nebraska Medical Center

Omaha, NE 68198-6025

Phone: 402-559-3790 (office), 402-559-2430 (lab)

Table S1: Summary of MS/MS parameters without 4-Phenyl-1,2,4-triazoline-3,5-dione (PTAD) derivatization: precursor ion, fragment ions, voltage potential (Q1), collision energy (CE) and voltage potential (Q3) for analytes.

| **Analyte** | **MRM (precursor/fragment ions)** | **Q1 (V)** | **CE (V)** | **Q3 (V)** |
| --- | --- | --- | --- | --- |
| 25(OH)D2 | 413.15>395.4 | -15 | -11 | -15 |
| 25(OH)D3 | 401.15>383.4 | -10 | -10 | -15 |
| Vitamin D2 | 397.3>69.1 | -19 | -25 | -13 |
| Vitamin D3 | 385.3>367.4 | -18 | -13 | -14 |
| d-3 vitamin D2 | 400.35>69.1 | -19 | -26 | -13 |
| d-6 25(OH)D3 | 407.2>389.4 | -19 | -11 | -15 |


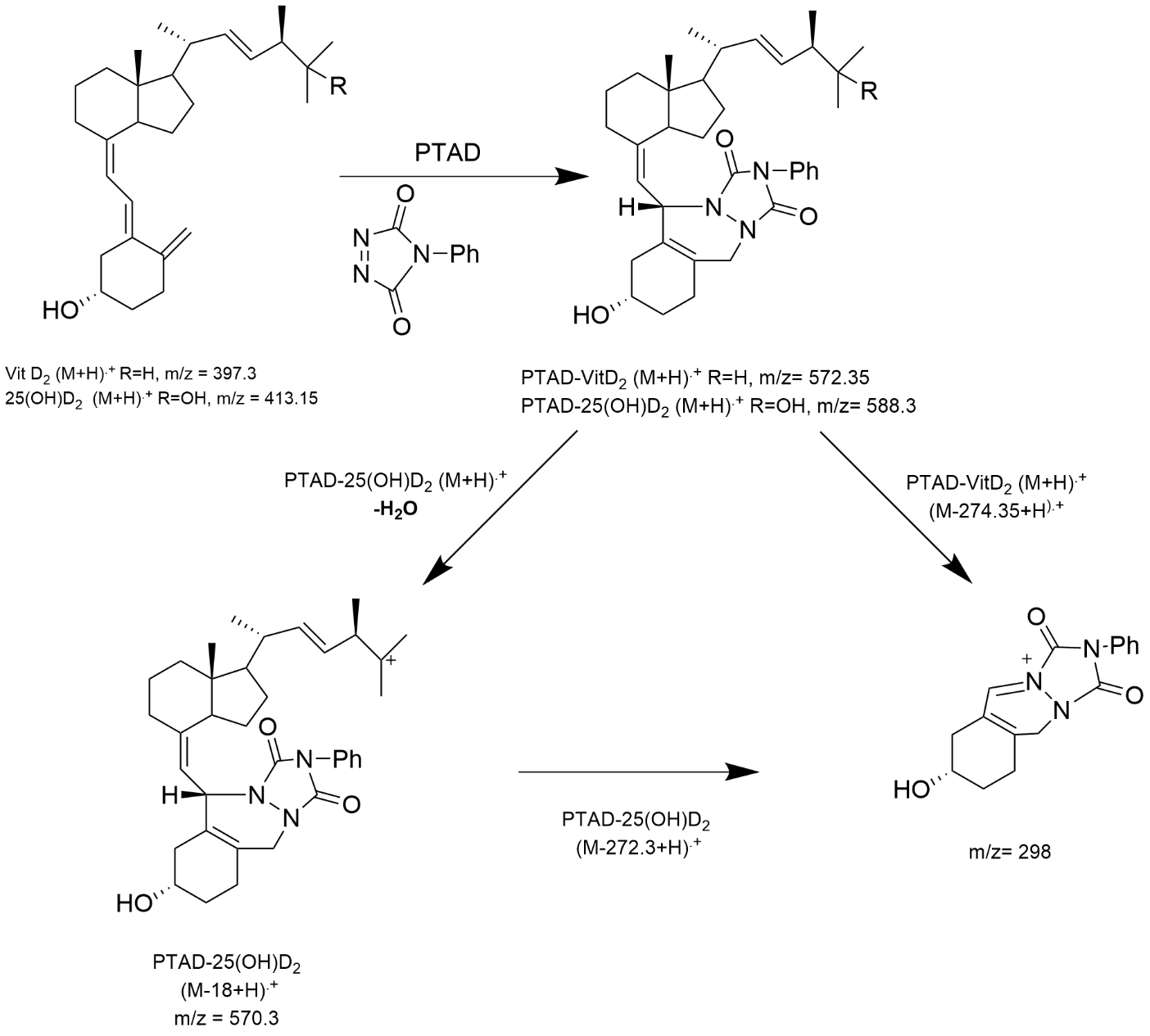


**Figure S1.** PTAD derivatization of vitamin D2 and 25(OH)D2 showing the PTAD-adduct ions at 572.35 and 588.3 respectively and the dehydrated precursor ions for 25(OH)D2 at 570.2.


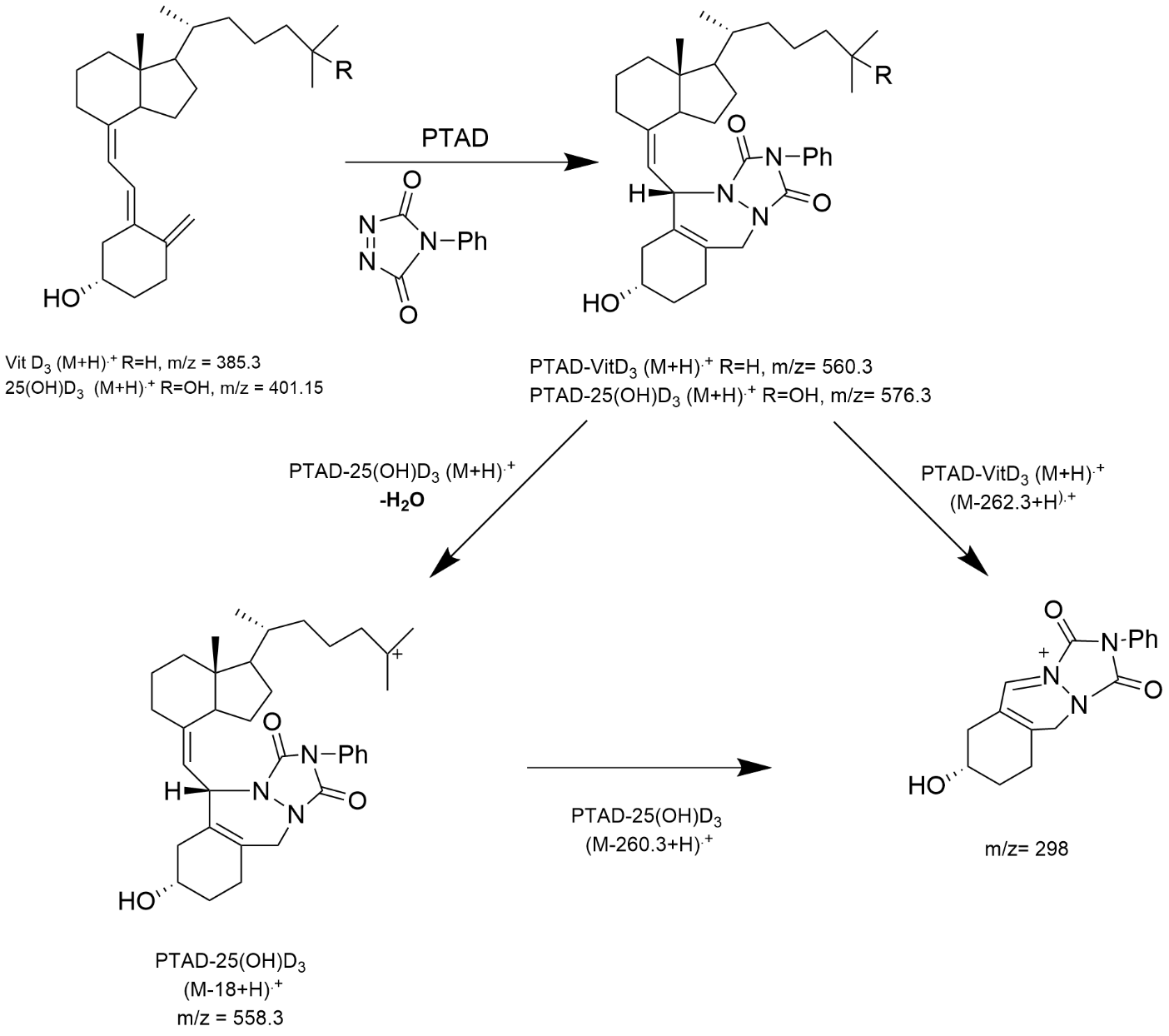


**Figure S2.** PTAD derivatization of vitamin D3 and 25(OH)D3 showing the PTAD-adduct ions at 560.3 and 576.3 respectively and the dehydrated precursor ions for 25(OH)D3 at 558.3.

Table S2: Summarizes the comparison of this method with previously reported LC-MS/MS methods related to the quantitation of vitamin D in Dried blood spots and human plasma.

| **Method** | **Analytes** | **Internal standard** | **Bio-matrix** | **Linearity (ng/mL)** | **Limit of detection**  **(ng/mL)** | **Run time**  **(min)** | **Ref.** |
| --- | --- | --- | --- | --- | --- | --- | --- |
| LC-ESI-MS/MS | 25(OH)D3 | d-6 25(OH)D3 | DBS | 2.25-225 | LOD: 5 | 10 | [26] |
| LC-API-MS/MS | 25(OH)D3  25(OH)D2 | d-3 25(OH)D3  d-3 25(OH)D2 | Serum | 2.5-100 | LLOQ: 2.5 | 7 | [37] |
| LC-ESI-MS/MS | 25(OH)D3  25(OH)D2 | d-6 25(OH)D3 | Serum | 1-100 | LLOQ: 1 | 3 | [38] |
| LC-ESI-MS/MS (This method) | 25(OH)D3  25(OH)D2  Vitamin D3  Vitamin D2 | d-6 25(OH)D3  d-3 vitamin D2 | DBS | 0.78-200 | LLOQ: 0.78 | 11 |  |

References

26. Lote-Oke, Rashmi, Jwala Pawar, Shriram Kulkarni, Prasanna Sanas, Neha Kajale, Ketan Gondhalekar, Vaman Khadilkar, Siddhesh Kamat, and Anuradha Khadilkar. "A Lc–Ms Method for 25-Hydroxy-Vitamin D3 Measurements from Dried Blood Spots for an Epidemiological Survey in India." *Scientific Reports* 10, no. 1 (2020): 19873.

37. Zhang, Stanley, Wenying Jian, Sheryl Sullivan, Banu Sankaran, Richard W. Edom, Naidong Weng, and David Sharkey. "Development and Validation of an Lc–Ms/Ms Based Method for Quantification of 25 Hydroxyvitamin D2 and 25 Hydroxyvitamin D3 in Human Serum and Plasma." *Journal of Chromatography B* 961 (2014): 62-70.

38. Lee, S., J. H. Kim, S. A. Kim, Y. S. Sun, A. Lee, S. J. Park, Y. T. Kim, K. R. Lee, and Y. J. Kim. "A Rapid and Simple Liquid-Chromatography-Tandem Mass Spectrometry Method for Measuring 25-Hydroxyvitamin D2 and 25-Hydroxyvitamin D3 in Human Serum: Comparison with Two Automated Immunoassays." *Ann Clin Lab Sci* 46, no. 6 (2016): 645-53.
