# Supplementary material for: A Simultaneous Extraction/Derivatization Strategy for Quantitation of Vitamin D in Dried Blood Spots Using LC–MS/MS: Application to Biomarker Study in Subjects Tested for SARS-CoV-2
Source: Int J Mol Sci. 2023 Mar 13;24(6):5489. doi: 10.3390/ijms24065489 (PMC10054002; doi:10.3390/ijms24065489)
Supplement: Supplementary file 1 [file ijms-24-05489-s001.zip › ijms-2210678-supplementary.pdf]

Supplementary Files

A simultaneous extraction/derivatization strategy for quantitation of Vitamin D in Dried blood spots using LC-MS/MS: Application to biomarker study in Subjects Tested for SARS-CoV-2

Yashpal S. Chhonker[1, #], Nusrat Ahmed[1, #], Christine M. Johnston[2], Ruanne Barnabas[3,4], Daryl J. Murry[1, 5, Δ]

[1]Clinical Pharmacology Laboratory, Department of Pharmacy Practice and Science, University of Nebraska Medical Center, Omaha, NE 68198, United States
[2]Department of Medicine, University of Washington, Seattle, WA
[3]Division of Infectious Diseases, Massachusetts General Hospital, Boston, MA
[4]Harvard Medical School, Boston, MA
[5]Fred and Pamela Buffett Cancer Center, University of Nebraska Medical Center, Omaha, NE 68198, United States.

[#] Authors contributed equally to this manuscript

[Δ] Corresponding author:
Daryl J. Murry, Pharm. D. (E-mail: dj.murry@unmc.edu)
Clinical Pharmacology Laboratory
Department of Pharmacy Practice and Science, College of Pharmacy
University of Nebraska Medical Center
Omaha, NE 68198-6025
Phone: 402-559-3790 (office), 402-559-2430 (lab)

Table S1: Summary of MS/MS parameters without 4-Phenyl-1,2,4-triazoline-3,5-dione (PTAD) derivatization: precursor ion, fragment ions, voltage potential (Q1), collision energy (CE) and voltage potential (Q3) for analytes.

**Analyte**	**MRM (precursor/fragment ions)**	**Q1 (V)**	**CE (V)**	**Q3 (V)**
25(OH)D2	413.15>395.4	-15	-11	-15
25(OH)D3	401.15>383.4	-10	-10	-15
Vitamin D2	397.3>69.1	-19	-25	-13
Vitamin D3	385.3>367.4	-18	-13	-14
d-3 vitamin D2	400.35>69.1	-19	-26	-13
d-6 25(OH)D3	407.2>389.4	-19	-11	-15

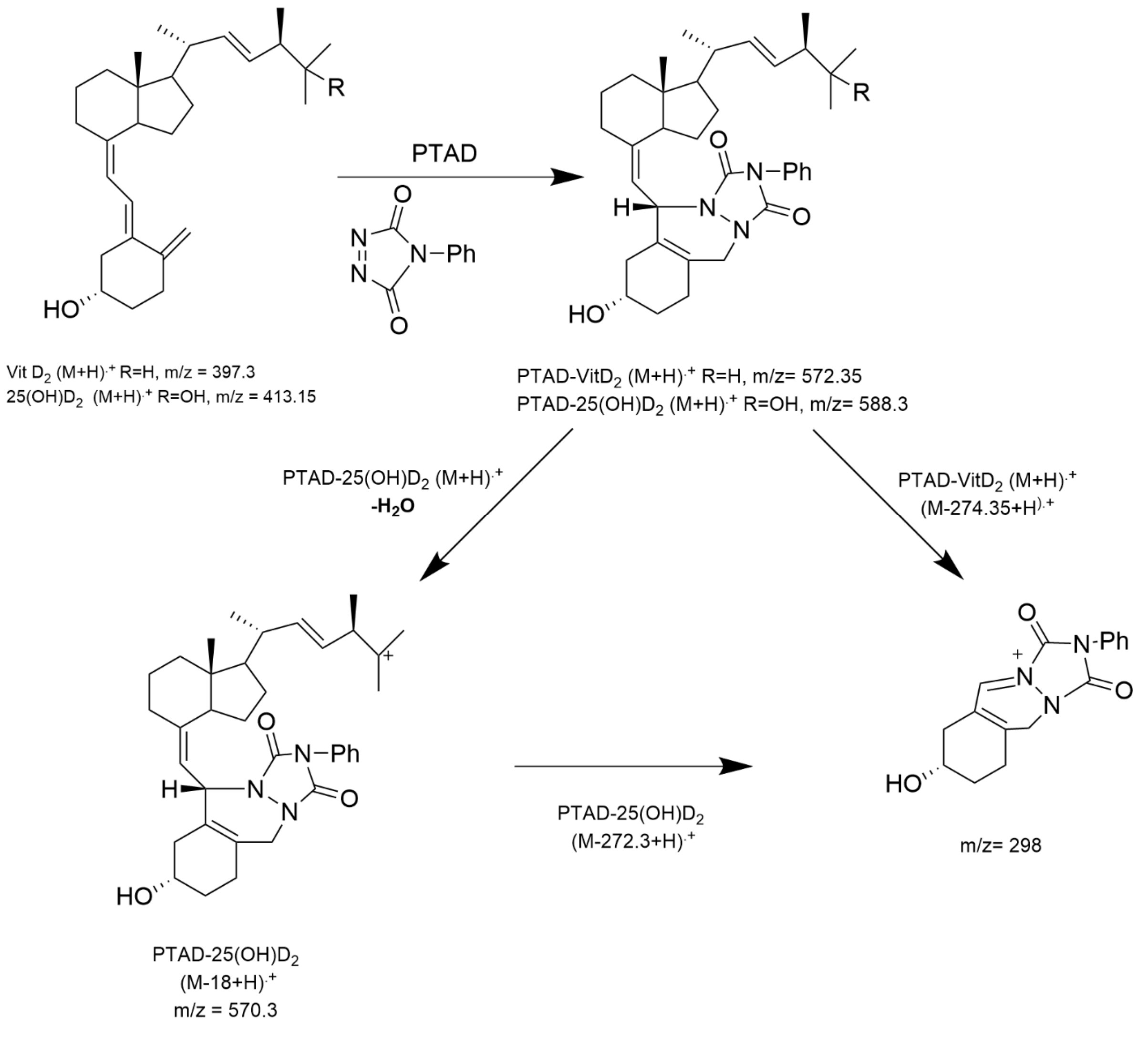

Figure S1. PTAD derivatization of vitamin D2 and 25(OH)D2 showing the PTAD-adduct ions at 572.35 and 588.3 respectively and the dehydrated precursor ions for 25(OH)D2 at 570.2.

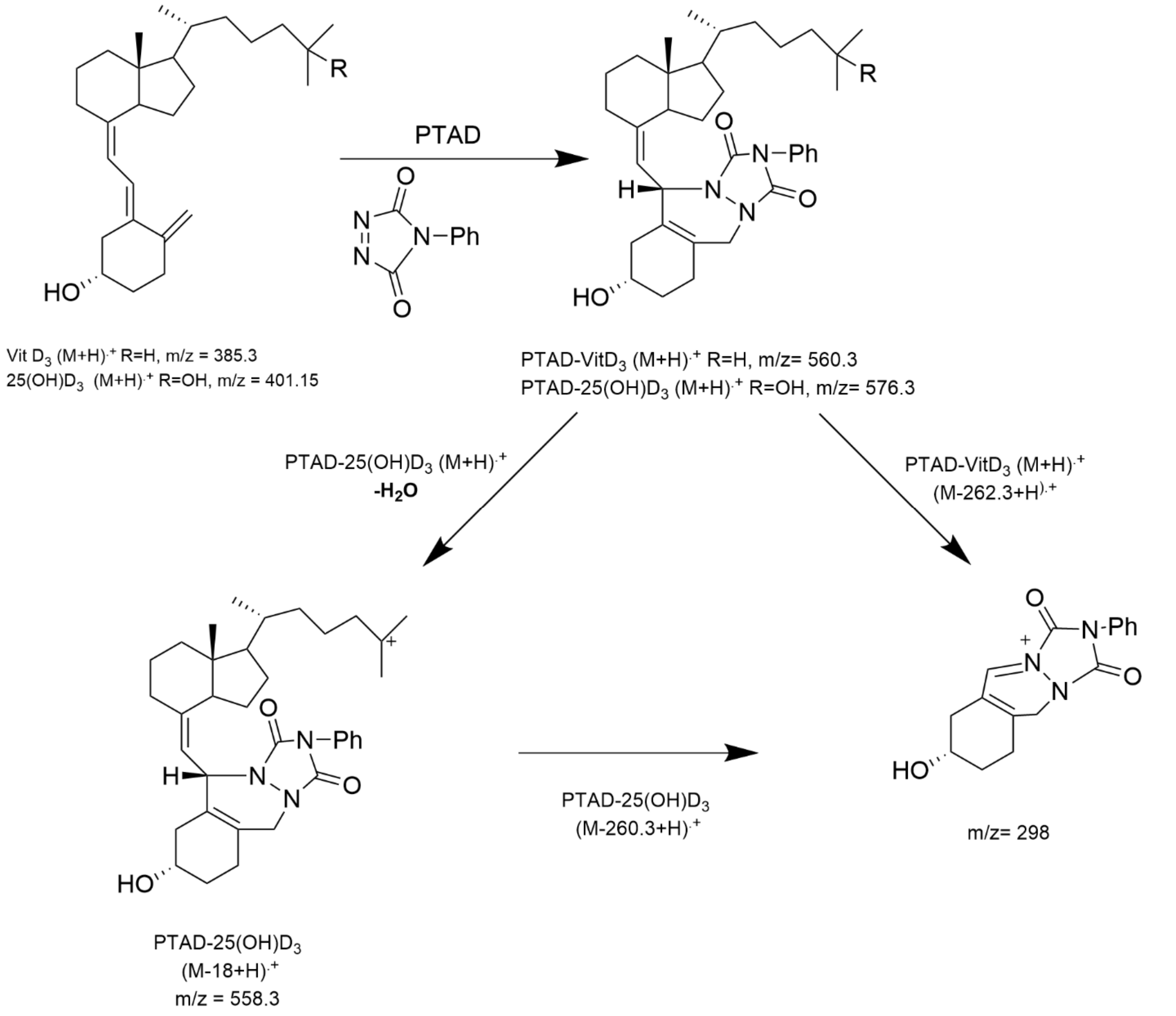

Figure S2. PTAD derivatization of vitamin D3 and 25(OH)D3 showing the PTAD-adduct ions at 560.3 and 576.3 respectively and the dehydrated precursor ions for 25(OH)D3 at 558.3.

Table S2: Summarizes the comparison of this method with previously reported LC-MS/MS methods related to the quantitation of vitamin D in Dried blood spots and human plasma.

Method	Analytes	Internal standard	Bio-matrix	Linearity (ng/mL)	Limit of detection (ng/mL)	Run time (min)	Ref.
LC-ESI-MS/MS	25(OH)D3	d-6 25(OH)D3	DBS	2.25-225	LOD: 5	10	[26]
LC-API-MS/MS	25(OH)D3 25(OH)D2	d-3 25(OH)D3 d-3 25(OH)D2	Serum	2.5-100	LLOQ: 2.5	7	[37]
LC-ESI-MS/MS	25(OH)D3 25(OH)D2	d-6 25(OH)D3	Serum	1-100	LLOQ: 1	3	[38]
LC-ESI-MS/MS (This method)	25(OH)D3 25(OH)D2 Vitamin D3 Vitamin D2	d-6 25(OH)D3 d-3 vitamin D2	DBS	0.78-200	LLOQ: 0.78	11	

References

26. Lote-Oke, Rashmi, Jwala Pawar, Shriram Kulkarni, Prasanna Sanas, Neha Kajale, Ketan Gondhalekar, Vaman Khadilkar, Siddhesh Kamat, and Anuradha Khadilkar. "A Lc–Ms Method for 25-Hydroxy-Vitamin D3 Measurements from Dried Blood Spots for an Epidemiological Survey in India." *Scientific Reports* 10, no. 1 (2020): 19873.

37. Zhang, Stanley, Wenying Jian, Sheryl Sullivan, Banu Sankaran, Richard W. Edom, Naidong Weng, and David Sharkey. "Development and Validation of an Lc–Ms/Ms Based Method for Quantification of 25 Hydroxyvitamin D2 and 25 Hydroxyvitamin D3 in Human Serum and Plasma." *Journal of Chromatography B* 961 (2014): 62-70.

38. Lee, S., J. H. Kim, S. A. Kim, Y. S. Sun, A. Lee, S. J. Park, Y. T. Kim, K. R. Lee, and Y. J. Kim. "A Rapid and Simple Liquid-Chromatography-Tandem Mass Spectrometry Method for Measuring 25-Hydroxyvitamin D2 and 25-Hydroxyvitamin D3 in Human Serum: Comparison with Two Automated Immunoassays." *Ann Clin Lab Sci* 46, no. 6 (2016): 645-53.
